# Relationship between tissue factor pathway inhibitor and aortic stiffness assessed by MRI: the Multi-Ethnic Study of Atherosclerosis (MESA)

**DOI:** 10.1186/1532-429X-18-S1-P353

**Published:** 2016-01-27

**Authors:** Yoshiaki Ohyama, Alban Redheuil, Bharath Ambale Venkatesh, Chikara Noda, Atul Chugh, Gisela Teixido-Tura, Jang-Young Kim, Chia-Ying Liu, Colin O Wu, Gregory Hundley, David A Bluemke, Joao A Lima

**Affiliations:** 1 Johns Hopkins University, Baltimore, MD USA; 2 Groupe Hospitalier La Pitié Salpêtrière Sorbonne Universités, Paris, France; 3 Jewish Hospital, Luisville, KY USA; 4 Vall d'Hebron Hospital, Barcelona, Spain; 5 Yonsei University, Seoul, Korea (the Republic of); 6 National Institutes of Health, Bethesda, MD USA; 7 National Heart, Lung and Blood Institute, Bethesda, MD USA; 8 Wake Forest School of Medicine, Winston-Salem, NC USA

## Background

Arterial stiffness is an important predictor of risk for incident cardiovascular events. Impaired endothelial function has been related to arterial cyclic stress, large artery stiffness and remodeling. MRI has been used to noninvasively measure local aortic strain and distensibility, and regional pulse wave velocity (PWV) of the proximal aorta. We investigated the relationship between aortic size and stiffness and tissue factor pathway inhibitor (TFPI) that is an endogenous inhibitor of the procoagulant tissue factor/factor VIIa complex, known as a marker of endothelial cell dysfunction in a large population free from overt cardiovascular disease.

## Methods

Multi-Ethnic Study of Atherosclerosis (MESA) participants with MRI parameters and TFPI measures at baseline (2000-2002) were studied. A phase contrast cine gradient echo sequence with ECG gating was performed to evaluate aortic size and through-plane flow. Images of the ascending and descending aorta were obtained in the transverse plane perpendicular to the aortic lumen at the level of the right pulmonary artery. Aortic sagittal oblique plane with black blood sequence was acquired to measure the distance from the ascending and descending aorta planes. Aorta analysis was performed using a validated automated software (ARTFUN, INSERM LIB). Brachial blood pressures were obtained during MRI. Linear regression models were used to evaluate the association between aortic size and aortic stiffness parameters and TFPI after adjusting for demographics and cardiovascular risk factors including age and systolic blood pressure.

## Results

A total of 552 participants (age 59.5 ± 9.5 years, 58.9% women, 44% White, 11% Chinese, 23% African American, 22% Hispanic) were included in this cross-sectional study. Table 1 shows descriptive statistics for aortic parameters according to TFPI quartile. As TFPI increased, aortic size increased and stiffness also increased, as demonstrated by decreased aortic strain/distensibility and increased PWV. In linear regression analysis, higher TFPI was independently associated with lower aortic strain and distensibility even after adjusting for demographics and cardiovascular risk factors (Table 2).

**Table 1:** Demographics and aortic measures according to TFPI quartile

	TFPI quartile				
	lowest			highest	
	Q1 (n = 140)	Q2 (n = 140)	Q3 (n = 143)	Q4 (n = 129)	p value
Age, years	57.7 (9.4)	58.2 (9.6)	60.0 (9.3)	62.2 (9.1)	<0.001
Male, %	25	44	50	45	<0.001
Systolic blood pressure, mmHg	121.4 (20.8)	121.7 (20.2)	124.2 (21.1)	130.9 (22.8)	<0.001
Diastolic blood pressure, mmHg	69.5 (10.3)	71.3 (8.7)	71.8 (11.0)	74.2 (10.3)	0.002
Pulse pressure, mmHg	51.9 (15.6)	50.4 (16.6)	52.3 (16.6)	56.8 (17.9)	0.01
TFPI, ng/ml	31.2 (5.5)	43.6 (2.9)	52.8 (2.7)	68.0 (9.5)	<0.001
Maximum aortic area, cm^2^	7.8 (1.7)	8.2 (1.9)	8.6 (1.9)	8.6 (1.9)	<0.001
Aortic strain, %	9.6 (6.8)	9.3 (7.0)	8.5 (4.8)	7.9 (5.5)	0.06
Aortic distensibility, mmHg^-1 ·10-3^	2.0 (1.7)	1.9 (1.4)	1.6 (1.1)	1.4 (1.0)	0.001
Pulse wave velocity, m/s	7.9 (4.6)	8.3 (5.1)	8.7 (5.5)	9.9 (6.8)	0.018

Values are represented as mean (SD) or %.

**Table 2:** The association between Aortic Indices and TFPI

	Model1	Model 2	Model3
Maximum aortic area, cm^2^	0.20 (0.06)*	0.08 (0.05)	0.05 (0.06)
Aortic strain, %	-0.46 (0.18)†	-0.38 (0.18)†	-0.42 (0.20)†
Aortic distensibility, mmHg^-1·10-3^	-0.16 (0.04)**	-0.11 (0.04)*	-0.10 (0.04)†
Pulse wave velocity, m/s	0.34 (0.16) †	0.14 (0.17)	0.19 (0.19)

Coefficients and SE (in brackets) were measured using multivariable linear regression models to assess the association of TFPI (per 10 ng/ml) with aortic measurements as dependent variables.

Model 1: Unadjusted.

Model 2: Adjusted for age, gender, and race.

Model 3: Model2 + BMI, heart rate, systolic blood pressure, LDL cholesterol, HDL cholesterol, hypertension medication use, diabetes mellitus, and smoking status.

**p < 0.001 *p < 0.01 †p < 0.05

## Conclusions

Our observations indicate that higher TFPI is independently related to reduced aortic strain and distensibility assessed by MRI, beyond the effects of age and cardiovascular risk factors including blood pressure. This suggests that increased local proximal aortic stiffness is independently associated with endothelial damage and dysfunction in humans free from overt cardiovascular disease.

